# Study protocol of the TEC-ORL clinical trial: a randomized comparative phase II trial investigating the analgesic activity of capsaicin vs Laroxyl in head and neck Cancer survivors presenting with neuropathic pain sequelae

**DOI:** 10.1186/s12885-022-10348-2

**Published:** 2022-12-05

**Authors:** Antoine Boden, Amélie Lusque, Sabrina Lodin, Marie Bourgouin, Valérie Mauries, Christelle Moreau, Amandine Fabre, Muriel Mounier, Muriel Poublanc, Nathalie Caunes-Hilary, Thomas Filleron

**Affiliations:** 1 Support Care Department, Institut Claudius Regaud IUCT-Oncopole, 1 avenue Irène Joliot Curie, 31059 Toulouse Cedex, France; 2 Biostatistics & Health Data Science Unit, Institut Claudius Regaud, IUCT-Oncopole, 1 avenue Irène Joliot Curie, 31059 Toulouse, France; 3 Clinical Trials Office, Institut Claudius Regaud, IUCT-Oncopole, 1 avenue Irène Joliot Curie, 31059 Toulouse, France

**Keywords:** Head and neck survivor, Neuropathic pain sequelae, Randomized phase II trials

## Abstract

**Background:**

Neuropathic pain is common in cancer survivorship and is one of the most distressing symptoms for patients previously treated for head and neck cancer. Persistent neuropathic pain, when it is ongoing and uncontrolled, has a detrimental effect and erodes patients’ quality of life. Patients treated for head and neck cancer are chronic opioid users to manage their post-treatment pain, which may entail an increased risk of addiction and overdose. We propose to evaluate the analgesic activity of high-concentration capsaicin patches for the treatment of head and neck cancer survivors presenting with neuropathic pain sequelae.

**Methods:**

TEC-ORL is a parallel, multicenter randomized comparative phase II study evaluating whether Capsaïcin patches (Qutenza®) reduce neuropathic pain when compared to Amitriptyline (Laroxyl®) in head and neck cancer survivors presenting with neuropathic pain sequelae. The primary efficacy outcome is the rate of patients with a pain reduction of at least two points at 9 months compared to baseline. Assuming that 5% of patients become lost to follow-up, 130 patients will need to be randomized to detect a 25% improvement (i.e., standard: 25%, experimental: 50%) using a one-sided chi-square test with an alpha of 0.05%. According to the recommendations for comparative phase II trials, the target differences and type I error rates are relaxed. Randomized patients will either be treated with a capsaicin 8% (Qutenza®) patch applied at three time intervals in the experimental arm or with Amitriptyline (Laroxyl®) (oral solution 40 mg/ml) taken for 9 months at the recommended daily dose of 25 mg to 75 mg in the control arm.

**Discussion:**

TEC-ORL is a randomized comparative phase II trial designed to comprehensively evaluate the analgesic activity of capsaicin compared to Laroxyl in Head and Neck Cancer survivors presenting with neuropathic pain sequelae.

**Trial registration:**

ClinicalTrials.gov identifier: NCT04704453 Date of registration: 2021/01/13.

## Background

After curative cancer treatment, pain may affect up to 40% of cancer patients in remission [1]. In the specific case of head and neck cancer, persistent pain remains a significant problem with 45% of survivors reporting pain and 10% severe pain [2]. Head and neck cancer treatment encompasses multiple therapies (surgery, chemotherapy and/or radiation) and may produce pain by damaging somatic tissues and nerves. The prevalence of persistent pain is similar for patients treated by surgery and/or radiotherapy [3]; and chemo-radiotherapy appears to increase the frequently of chronic pain [4]. For patients treated by chemo-radiotherapy, pain is predominantly of neuropathic origin which is defined as pain caused by a lesion or disease of the somatosensory nervous system and associated with prominent symptoms such as burning, electric shock, tingling and itching sensations [5]. Persistent and uncontrolled neuropathic pain, has a detrimental effect and erodes patients’ quality of life. The pathophysiological intricacies of neuropathic pain are yet to be fully understood; its management is multifaceted and remains a challenge [6]. Treatment algorithms are distinct from those for nociceptive pain and are based on non-opioid and opioid treatments [6, 7]. Guidelines recommend amitriptyline as a first-line treatment for neuropathic pain (neoplastic or non-neoplastic) [6, 7] and for the sequalae of head and neck pain [8]. Recently, Pregabalin demonstrated a significant analgesic benefit compared to placebo in patients with head and neck cancer with radiotherapy-related neuropathic pain [9]. Pegabrilinin is yet to be adopted as a standard of care for neuropathic pain as it treats fewer symptoms than tricyclic antidepressants. Notwithstanding the efficacy of first-line therapy, some patients continue to suffer from uncontrolled neuropathic pain with only weak recommendations supporting the second-line use of lidocaine patches, high-concentration capsaicin patches, and tramadol; and a weak recommendation for strong opioids and botulinum toxin, as a third-line treatment option [6].

A recent study highlights that 50% of patient’s treated for head and neck cancer are chronic opioid users to manage their post-treatment pain [10], which may entail an increased risk of addiction and overdose [11]. It therefore seems appropriate to improve first-line treatment efficacy to spare the use of opiods in favor of other mechanisms of action. High-concentration capsaicin patches (Qtenza®) are an efficient treatment for neuropathic pain [12] and have been proposed as a second-line treatment after the failure of tricyclic antidepressant or antiepileptic treatment [6]. Caspaicin is the main active ingredient found in hot chili peppers. Pharmacologically, it is a potent and highly selective Transient Receptor Potential Vanilloid-1 (TRPV1) agonist. TRPV1 is a polymodal nociceptor which play an important role in detecting a number of pain stimuli (heat, acids etc.) [13]. Qtenza@ activates TRPV1 expressing nociceptors which cause the onset of pain and erythema. Using a process described as ‘defunctionalization’ of nociceptor fibers, cutaneous hypersensitivities are then attenuated and pain reduced [14]. Despite these interesting properties, high-concentration capsaicin patches are not recommended to treat head and neck pain. In fact, neck and face application may be associated with adverse effects (erythema, pain, irritation) due to accidental mucosa exposure. Several publications do however highlight the feasibility and potential efficacy of capsaicin treatment in this particular setting [15, 16].

Given that capsaicin treatment of the head and facial region has proven to be feasible albeit with specific warning and precaution caveats, our hypothesis is that high-concentration capsaicin patches reduce neuropathic pain when compared to Amitriptyline (Laroxyl®) in head and neck cancer survivors presenting with neuropathic pain sequelae.

## Methods and design

### Trial objectives

#### Primary objective

The primary objective of this study is to evaluate whether Capsaïcin patches (Qutenza®) reduce neuropathic pain when compared to Amitriptyline (Laroxyl®) in head and neck cancer survivors presenting with neuropathic pain sequelae.

#### Secondary objectives

Secondary objectives include a sensitivity analysis, the evolution of neuropathic pain, the safety and health related quality of life.

### Trial design

This is a Phase II, multicenter, randomized open-label and comparative study designed to evaluate whether Capsaïcin patches (Qutenza®) reduce neuropathic pain when compared to Amitriptyline (Laroxyl®) in head and neck cancer survivors presenting with neuropathic pain sequelae (ClinicalTrials.gov Identifier: NCT04704453). The primary endpoint is pain evaluated using a numeric scale. We opted for a randomized comparative phase II trial design rather than a single arm or a randomized non-comparative phase II trial to avoid obtaining false positive or false negative results only related to the study hypothesis [17]. All head and neck cancer patients presenting with neuropathic pain sequelae will be eligible for inclusion into the study. Patients that give their informed consent will be enrolled and randomized into the Amitriptyline (Laroxyl®) or Capsaïcin patch (Qutenza®) arm of the study.

### Clinical study endpoints

#### Primary endpoint

The primary efficacy outcome is the rate of patients presenting a pain reduction of at least two points at 9 months compared to baseline. Pain will be evaluated using a numeric scale from 0 (No pain) to 10 (Worst pain) by considering the average pain in the last 24 hours. Modification of a pain treatment strategy will be considered as a failure if the modification relates to neuropathic pain. Pain treatment modifications that are not related to neuropathic pain and missing values will not be considered as a failure.

#### Secondary endpoints

The sensitivity endpoint is defined as the rate of patients presenting a pain reduction of at least two points at 9 months compared to baseline. Modification of the pain treatment strategy for any reason or patients with missing values will be scored as a failure*.

Neuropathic pain will be evaluated using the French language Neuropathic Pain Symptom Inventory (NPSI) which was developed and validated in that language [18]. The NPSI is a self-administered questionnaire specifically designed to evaluate the different symptoms of neuropathic pain.

Safety will be assessed using the toxicity grading of the National Cancer Institute (NCI-CTCAE v5).

Quality of life will be evaluated with the EORTC QLQ-C-30 questionnaire.

### Randomization

After giving their written informed consent, patients which satisfy all the inclusion and non-inclusion criteria (Table 1) will be randomized by the sponsor at a 1:1 ratio to one of the two study arms. Randomization will be stratified according to: patient center, neuropathic pain at baseline (5 vs > =5), analgesic treatment at inclusion (no analgesic or neuropathic pain opioid treatment vs opioid treatment vs non-opioid treatment for neuropathic pain). A dynamic randomization procedure by minimization will be used. Randomization will be performed centrally by the IUCT-O clinical trials office using the TENALEA Clinical Trial Data Management System (online secure internet).

**Table 1:** Inclusion and non-inclusion criteria

Inclusion Criteria	Non-Inclusion criteria
- Age ≥ 18 years. - HN cancer in remission: absence of clinical or radiological signs of progression at least 3 months after specific treatments. - Pain of the cervico-facial sphere persisting for more than 3 months after surgical and/or radiotherapy treatment. - Peripheral neuropathic character of pain objectified as a score ≥ 4/10 on the DN4 questionnaire. - Average pain intensity over the past 24 hours assessed on the numerical scale as ≥2/10. - Postmenopausal patient or patient who agrees to use effective contraception for the duration of treatment and for a minimum of 15 days after the end of the treatment period. Non-menopausal patients must have a negative pregnancy test prior to inclusion in the study. - Patient affiliated to a Social Health Insurance in France. - Patient who signed informed consent prior to inclusion in the study and prior to any specific study procedures.	- HN cancer progression. - Other concomitant neoplasia (progressive or not). - Central pain etiology. - Average pain intensity over the past 24 hours is assessed on the numerical scale as < 2/10. - Allergy to any of the components of the capsaicin patch. - Capsaicin patch cannot be applied to the area to be treated despite taking the precautions described in the protocol because of proximity to mucous membranes or eyelids. - Contraindication of amitriptyline treatment. - Patient with an unhealed skin lesion on the area to be treated. - Previous course of capsaicin or amitriptyline treatment. - Topical treatment of the painful area used for more than 21 days before inclusion. - Ongoing opioid treatment > 80 mg/day oral morphine equivalent. - Uncontrolled high blood pressure or cardiovascular history (infarction, stroke, pulmonary embolism) less than 3 months ago. - Patient included in another interventional therapeutic trial. - Pregnant or breastfeeding patient. - Any psychological, family, geographical or sociological condition that prevents compliance with the medical follow-up and/or procedures of the study protocol. - Patient who has forfeited his/her freedom through an administrative or judicial sentence or who is under legal custody (curatorship and guardianship, protection of justice).

### Treatment

#### Standard of care

Patients randomized to the control arm will be treated with Amitriptyline (Laroxyl®) (oral solution 40 mg/ml) taken for 9 months at the recommended daily dose of 25 mg to 75 mg.

#### Experimental treatment

Patients randomized to the experimental arm will be treated by application of an 8% capsaicin patch (Qutenza®) at three time intervals: the first application within two weeks of randomization and the next two applications at three monthly intervals.

### Follow-up

Patients will be followed-up every 3 months from the date of randomization. Assessments will include, clinical examination, pain evaluation using the numeric scale (primary endpoint), quality of life evaluation based on the EORTC QLQ-C30 questionnaire and neuropathic pain evaluation with the NPSI. Any adverse events (AE) related to the study treatments will be collected until completion of the study.

#### Data collection

Patient data will be collected by the investigator (or representatives) via an electronic Care Report Form (e-CRF) provided by the Institut Claudius Regaud Data Management Department. The e-CRF is based on ENNOV Clinical® software edited by Ennov Company. It is available on the secure website. Study data validation will be carried out to verify the completeness, the accuracy and consistency of data collected in the e-CRF. Data clarification forms will be issued by the Data Management Department of ICR in order to allow investigators sites to resolve data deficiencies and/or in accuracy.

### Sample size calculation

Based on the literature, we expect 25% of patients to present with a pain decrease of at least two points [6]. To detect a 25% improvement (i.e., standard: 25%, experimental: 50%), using a one-sided chi-square test with an alpha of 0.05%, will require the enrollment of 125 patients, for 90% power. Assuming that 5% of patients will be lost to follow-up, we will need to randomize 130 patients (Randomization ratio1:1). According to the recommendations for comparative phase II trials, the target differences and type I error rates are relaxed.

### Main statistical analysis

The primary endpoint will be analyzed on the Intent To Treat population and expressed as values and percentages with 95% one-sided confidence intervals. Treatment effect will be estimated using a logistic regression model adjusted for stratification factors [19]. Odds ratios will be estimated with their 95% confidence intervals (one sided). The sensitivity analysis will be conducted using a similar method. Depending on the extent of missing data, the following complementary analyses may be conducted to evaluate its influence on the results of the study: case complete study, multiple imputation for missing data.

## Discussion

As the population of head and neck cancer survivorship increases, it has become increasingly important for health care providers to propose an optimized treatment for neuropathic pain. There is an unmet need for well-designed, robust clinical trials to help improve therapeutic strategies in this setting. Single arm trials, introduce several forms of bias including patient selection, and misspecification of the hypothesis. We therefore opted for a randomized comparative phase II TEC-ORL trial design. Randomized comparative trials are an essential tool to evaluate therapeutic strategies and new treatments. However, designing and conducting a randomized trial on supportive care for neuropathic pain is associated with several challenges [20]. Several new treatments that appeared effective in phase 2 trials subsequently failed to confirm efficacy in phase 3 registration trials. This may be explained by the application of restrictive inclusion criteria in the phase 2 trial to maximize treatment effect differences, but this approach ultimately impedes the external validity, and/or generalizability of the phase 2 trial. Restricted inclusion criteria may also potentially limit the relevance of randomized controlled trial results to ‘real-world’ practices and make patient recruitment difficult [21]. The primary reason for integrative pain trial failure is inadequate patient recruitment numbers. As cancer pain management takes place in the context of multiple, sometimes competing personal interests, the primary reason for non-participation is due to the patient and/or investigator. The other challenge is the poor retention of participants in pain trials, which may lead to missing outcome data and may introduce bias and reduce study power. In this setting, there is a need for specific strategies to improve the recruitment and retention of participants in randomized trials.

Neuropathic pain is common in cancer survivorship and is one of the most distressing symptoms for patients previously treated for head and neck cancer. Neuropathic pain also decreases quality of life. There is a need to provide optimal first-line treatment to reduce neuropathic pain and to limit chronic opioid use for post-treatment neuropathic pain management [10]. To the best of our knowledge, TEC-ORL is the first randomized comparative phase II trial investigating the activity of high-concentration capsaicin patches as a first-line therapy for head and neck cancer survivors presenting with neuropathic pain sequelae.

## Data Availability

Data sharing is not applicable to this article as no datasets were generated or analyzed during the current study.

## Abbreviations

NCI: National Cancer Institute
CTCAE: Common Terminology Criteria for Adverse Events
TRPV1: Transient Receptor Potential Vanilloid-1
NPSI: Neuropathic Pain Symptom Inventory
EORTC: European Organisation for Research and Treatment of Cancer
QLQ-C30: Quality of life Questionnaire Core 30
AE: Adverse event
